# Using Ancient Traits to Convert Soil Health into Crop Yield: Impact of Selection on Maize Root and Rhizosphere Function

**DOI:** 10.3389/fpls.2016.00373

**Published:** 2016-03-30

**Authors:** Jennifer E. Schmidt, Timothy M. Bowles, Amélie C. M. Gaudin

**Affiliations:** ^1^Department of Plant Sciences, University of California at DavisDavis, CA, USA; ^2^Department of Natural Resources and the Environment, University of New HampshireDurham, NH, USA

**Keywords:** crop breeding, domestication, maize (*Zea mays*), microbiome, resource acquisition, rhizosphere, roots, soil health

## Abstract

The effect of domestication and modern breeding on aboveground traits in maize (*Zea mays*) has been well-characterized, but the impact on root systems and the rhizosphere remain unclear. The transition from wild ecosystems to modern agriculture has focused on selecting traits that yielded the largest aboveground production with increasing levels of crop management and nutrient inputs. Root morphology, anatomy, and ecophysiological processes may have been affected by the substantial environmental and genetic shifts associated with this transition. As a result, root and rhizosphere traits that allow more efficient foraging and uptake in lower synthetic input environments might have been lost. The development of modern maize has led to a shift in microbiome community composition, but questions remain as to the dynamics and drivers of this change during maize evolution and its implications for resource acquisition and agroecosystem functioning under different management practices. Better understanding of how domestication and breeding affected root and rhizosphere microbial traits could inform breeding strategies, facilitate the sourcing of favorable alleles, and open new frontiers to improve resource use efficiency through greater integration of root development and ecophysiology with agroecosystem functioning.

## Introduction

**Figure 1:**
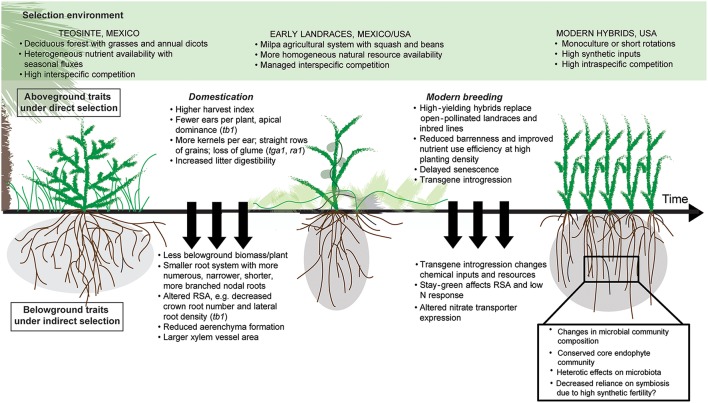
**Factors driving shifts in maize root and rhizosphere function**. Substantial changes in environment from wild forests to managed agroecosystems, combined with selection on aboveground traits, has resulted in shifts in maize root and rhizosphere function. The timing of altered nitrate transporter expression is unclear, and questions remain about the extent of effects on the rhizosphere microbiome.

Since its origin in the Balsas river valley of present-day Mexico 10,000 years ago, maize has undergone dramatic changes in shoot development and physiology as early agriculturists and modern breeders selected for greater yield response to increasingly managed agroecosystems (Harlan et al., 1973). Teosinte (*Zea mays* ssp. *parviglumis*), the ancestor of modern maize, originates from a mountainous environment with seasonal nutrient fluxes and high interspecific competition with diverse deciduous trees, grasses, and annual dicots (Gaudin et al., 2011b). After domestication around human settlements in fertile alluvial river banks, early maize varieties spread to other parts of Americas, where landraces were cultivated in traditional *milpa* agricultural systems (maize-bean-squash intercropping; Zizumbo-Villarreal and Colunga-GarciaMarin, 2010). Subsequent innovations following the industrial revolution such as mechanized tillage and the replacement of crop residues and organic inputs with synthetic fertilizers altered the agricultural landscape substantially, creating the homogeneous, nutrient-rich, high-intraspecific-competition environment seen in present-day monocultures and short rotations (Figure 1).

Here we argue that directed selection pressure for yield and aboveground traits during maize evolution coupled with shifts toward high-input, high-density selection environments may have inadvertently altered root system development and ecophysiological functioning. Thus, both host-genotype-driven changes in the ability of maize to recruit and respond to microbial interactions and environment-driven selection pressure on integrated plant and microbial functions may have altered coevolution of the microbiome (Kiers and Denison, 2014; Vandenkoornhuyse et al., 2015; Figure 1). Root and rhizosphere interactions have traditionally been neglected in discussions of maize domestication and breeding, despite their importance for plant fitness and productivity at lower input levels. The transition from wild ecosystems to modern maize monocultures may also have altered the ability of roots to dynamically respond to changes in resource availability, cope with stress and rely on microbial interactions in the rhizosphere to cycle and acquire soil resources (Wissuwa et al., 2009; Zancarini et al., 2012), which are essential functions in biologically-based and low input systems.

While past intensification of agriculture dramatically increased crop yields, future increases in productivity required for a growing population must come at a lower environmental cost. For instance, low nutrient use efficiencies and subsequent nutrient losses, especially of nitrogen (N) and phosphorus (P), contribute to eutrophication, climate forcing, and loss of biodiversity, with serious impacts on human and ecosystem health (Robertson and Vitousek, 2009). Further, climate change will cause more variability in precipitation and temperature (Kirtman et al., 2013) with consequences for crop growth, nutrient cycling, and yields (Lobell et al., 2014). Shifting to more biologically-based or lower input cropping systems shows promise for sustaining or increasing yields while reducing environmental costs and also increasing resilience to extreme events (Bommarco et al., 2013). But if crops are not well-adapted to these new agro-environments, then yield potential may not be fully realized.

**Figure 2:**
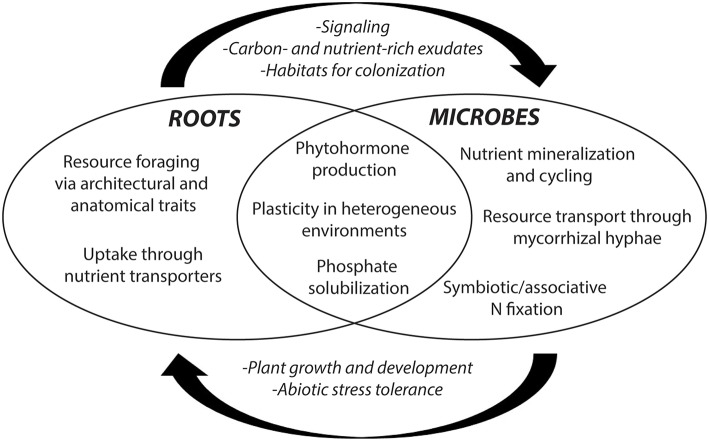
**Rhizosphere interactions between root- and microbe-specific traits in resource acquisition**. Resource acquisition is facilitated by roots, microbial symbionts, and/or interplay between the two. Rhizosphere interactions are of particular importance for resource acquisition in biologically-based systems that rely on microbial nutrient cycling rather than external inputs.

Investigating the extent and significance of inadvertent changes belowground during the course of artificial selection is highly relevant to support crop breeders in developing maize varieties able to take full advantage of microbial interactions and high rates of nutrient cycling created by soil-health building management practices. While development and plasticity of root system architecture and physiological traits enable foraging and uptake of soil resources, rhizosphere ecology facilitates plant resource acquisition through synergisms with microbes and exudate production (Figure 2). As such, roots and rhizosphere interactions could prove key to developing sustainable maize production systems (Bishopp and Lynch, 2015).

This review examines scientific evidences and potential drivers of changes in maize root morphology, anatomy, and physiology from teosinte through early landraces to modern hybrids and considers their functional significance for resource acquisition. We discuss current knowledge of human selection-driven shifts in maize rhizosphere ecology in light of the underlying plant-driven (G), environment-driven (E), and genotype-by-environment (G × E) mechanisms and highlight research gaps to be addressed in the future.

## Have shoot and root traits co-evolved?

### Morphological traits

The suite of traits selected during the domestication of crop wild relatives to increase yield or facilitate agronomic cultivation are collectively described as “domestication syndrome” (Hammer, 1984). Domestication traits commonly include enhanced fruit production, altered vegetative shoot morphology, and changes in secondary metabolites (e.g., decreased bitterness; Meyer et al., 2012). In cereals, domestication has led to increased seed size and number, increased apical dominance, changes in photoperiodicity, loss of seed dormancy, changes in grain composition, and loss of seed shattering (Harlan et al., 1973; Gross and Olsen, 2010; Abbo et al., 2014).

Aboveground morphological differences between teosinte and modern maize are striking. Teosinte has clusters of small ears in the axils of multiple leaves per stem, whereas modern cultivars show increased biomass and apical dominance with one ear per node and fewer than two ears per stem. Maize ears contain hundreds of large, naked kernels as compared to the few, small, glume-encased kernels of teosinte ears (Harlan et al., 1973). These changes are mostly attributed to the domestication genes *teosinte branched1* (*tb1*) and *barren stalk1* (*ba1*) (Doebley et al., 1997; Gallavotti et al., 2004; Hufford et al., 2007, 2012), controlling vegetative meristem development, *teosinte glume architecture1* (*tga1*), accounting for the naked kernels (Dorweiler et al., 1993; Wang et al., 2005), and *ramosa1* (*ra1*), controlling kernel row regularity (Dempewolf, 2010; Sigmon and Vollbrecht, 2010).

While root traits were likely not under intentional selection during domestication, plants usually respond to changes in shoot size by compensatory changes in root growth and architecture (Gaudin et al., 2014), perhaps to maintain balance between resource sinks and source tissues. As such, root morphology may have been altered indirectly by selection for higher harvest index and related traits such as apical dominance. In comparison to early landraces, teosinte (*ssp* parviglumis) has fewer seminal roots, possibly related to smaller seed size, but a greater number of narrower, shorter, more branched nodal roots, which may be beneficial for early P acquisition (Burton et al., 2013). Domestication studies have also shown common genetic control of above- and belowground morphology as decrease in *tb1* function in modern maize restores the teosinte phenotype both above and belowground, resulting in a larger root system with numerous and highly branched crown roots (Gaudin et al., 2014). Because of their impact on sink strength and nutrient demand, other known domestication genes may also show correlated belowground effects.

Following domestication, human selection for desirable traits has continued to affect maize shoot and root morphology over centuries of landrace cultivation and decades of inbred and hybrid breeding. Desirable improvement traits have included tolerance to higher planting densities (Duvick, 2005) as well as abiotic stresses such as drought and heat (Tollenaar and Lee, 2002; Campos et al., 2006), the introduction of delayed senescence (stay-green) (Lee and Tollenaar, 2007), and resistance to biotic stresses such as insect herbivory and weed competition via introgression of transgenes (Box 1). Although R:S ratio remained conserved across 9000 years of breeding and selection (Gaudin et al., 2011a), modern stay-green hybrids have greater total root length and deeper roots than their non-stay-green counterparts (Ning et al., 2014). As a result of breeding in inorganic nutrient-saturated and homogeneous environments with high intraspecific competition, the root systems of more recent maize cultivars have shallower root angles, fewer nodal roots, and greater distance from nodal roots to lateral branching, potentially enhancing deep resource foraging and minimizing root system overlap (York et al., 2015). The tradeoffs of increased investment in deep roots should be investigated, for example to determine whether corresponding decreases in the lateral roots that are preferentially colonized by arbuscular mycorrhizal (AM) fungi (Gutjahr and Paszkowski, 2013) have affected benefits of AM colonization. Collectively, these reports suggest that domestication and breeding may have decreased topsoil foraging ability and mycorrhizal colonization sites of single plants while increasing exploration of deeper soil layers by plant populations.

Box 1 **Transgenes and the Maize Rhizosphere**

Introgression of genetic material from other species has contributed substantially to modern maize productivity and as such deserves attention here despite being a fundamentally different form of genetic modification than traditional breeding. Modern commercial maize hybrids possess up to eight stacked transgenes, most frequently conferring tolerance to the herbicides glyphosate or glufosinate or resistance to insect pests such as corn rootworm, coleopterans, and corn root borer (Dunwell, 2014). Although a detailed impact assessment of these transgenes on the rhizosphere is outside the scope of this review, the topic deserves attention in a discussion of modern maize.

Transgenes are predicted to affect the rhizosphere microbiome primarily through chemical inputs and altered resource provisioning. Introgression of herbicide tolerance, found in almost all modern hybrids (Dunwell, 2014), is accompanied by inputs of the corresponding chemical. Glyphosate alters rhizobacterial community composition and increases the prevalence of pathogenic *Fusarium* (Kremer and Means, 2009), but appears to have less far-reaching impacts than an alternative pre-emergence herbicide containing acetochlor and terbuthylazine (Barriuso et al., 2010). However, another study found no effect of glyphosate-resistant maize or glyphosate application on denitrifying bacteria or the fungal community in the rhizosphere (Hart et al., 2009). *Bt* maize affects rhizosphere resource availability. *Bt* maize differs in lignin content and root exudate composition from its non-transgenic counterpart (Saxena et al., 1999; Saxena and Stotzky, 2000, 2001). However, greenhouse and field studies have shown no difference in population size, metabolic profile, or genetic diversity of rhizobacteria (Brusetti et al., 2004; Fang et al., 2005; Icoz et al., 2008; Prischl et al., 2012; Bumunang and Babalola, 2014) although genetic analysis has found some differences (Brusetti et al., 2004). Ecological functions such as nutrient cycling may nonetheless be affected, given that *Bt* introgression influences abundances of archaea and bacteria involved in N metabolism (Cotta et al., 2014). Mycorrhizal community composition (Tan et al., 2011) and spore density (Cheeke et al., 2014), but not but colonization potential (Tan et al., 2011), appear to be affected by host *Bt* status.

Transgenes have improved maize productivity and recent studies have introgressed genes improving abiotic stress tolerance, nutrient use efficiency, and nutritional quality of maize (Dunwell, 2014). However, given the potential for transgenes to affect the rhizosphere microbiome and its vital role in ecological function through altered inputs and resource quality, new transgenes should be carefully evaluated for rhizosphere impacts in addition to existing risk assessments.

### Anatomical developments

Comparisons between teosinte, landraces and modern varieties have revealed major differences in root anatomical traits involved in resource acquisition and transport. Teosinte has higher rates of cortical aerenchyma formation, which can reduce the metabolic cost of roots under stressed conditions, and greater phenotypic variation for this trait than landraces (Burton et al., 2013). Modern varieties have lower aerenchyma plasticity as compared to teosinte, which forms aerenchyma constitutively under non-stressed conditions as well as in response to stress (Mano et al., 2007). This suggests that anatomical traits mediating adaptation to drought and nutrient-limited conditions (Lynch, 2007) may have been selected against in the irrigated, fertilized environment of modern maize agroecosystems. However, larger total xylem vessel area (XVA) in landraces and modern cultivars as compared to teosinte (Burton et al., 2013) may provide quicker transport of water and nutrients in higher-resource environments to meet the demands of a larger shoot system, perhaps at the cost of increased cavitation under drought stress (Tyree et al., 1994). Since XVA alone is not necessarily related to hydraulic conductivity (Smith et al., 2013), further analysis is needed to determine if the increase in total XVA is formed by larger-diameter vessels, which would increase flow rates (Tyree and Ewers, 1991). Newer modern hybrids have smaller but more numerous xylem vessels as compared to older modern hybrids (York et al., 2015), but vessel size in teosinte and landraces remains to be measured.

### Root physiological attributes

Along with shifts in architectural and anatomical traits, root physiological activity may have been altered to accompany increases in crop N and water demand during the growing season (Antonietta et al., 2015) to support more vigorous vegetative growth, increased sink capacity during grain filling (Lee and Tollenaar, 2007), and delayed senescence. For instance, two stay-green hybrids had higher N uptake during grain filling (He et al., 2003) and a larger kernel response to N availability than their non-stay-green counterparts (Antonietta et al., 2015). However, scarce information is available on how kinetics and regulation of water and nutrient uptake changed during maize evolution. Productivity gains seen in newer hybrids result in part from increased N (York et al., 2015) acquisition and water (Reyes et al., 2015) use efficiencies compared to earlier varieties; however, evidence conflicts as to whether water and total N uptake has increased over time (Hammer et al., 2009; Nagore et al., 2014; Reyes et al., 2015). Resistance to N stress, as measured by no effect of low N availability on root or shoot biomass, in two teosintes was attributed in part to differential regulation of genes involved in N assimilation and metabolism as compared to five modern maize lines, although regulation of three N metabolism genes was consistent across all seven lines (Han et al., 2015). Teosinte possesses orthologs of four of the seven modern maize nitrate transporter genes, but upregulates expression of *ZmNrt2.3*, involved in the high-affinity system, twice as much as modern maize under low N conditions (Gaudin et al., 2011b). These physiological attributes may have changed the kinetics of N uptake, particularly under conditions of low soil ${\text{NO}}_{3}^{-}$ levels that are common in biologically-based systems, making modern maize better-suited to high inorganic N environments with a corresponding decrease in adaptability to low-input agroecosystems (Ruzicka et al., 2012). Possible changes in ammonium transporters and assimilation should be further studied, as ammonium may be a more significant source of N in lower-input or biologically based soils (Burger and Jackson, 2003).

### Root plasticity

Root plasticity allows plants to adjust root system architecture in response to changing resource availability. Selection pressure on root phenotypic plasticity is particularly relevant to breeding for rhizosphere traits involved in nutrient use efficiency. Schlichting (1989) proposed that the evolution of plasticity would be favored by heterogeneous environments, but it does not appear that the converse has occurred. Homogeneous breeding conditions have not triggered a loss of plasticity and modern maize remains capable of a plastic response to heterogeneous nitrogen supply (Yu et al., 2014), although the particular mechanisms of this plastic response have changed. Teosinte responds to low-nutrient environments and shade by decreasing shoot tillering to reduce nutrient requirements, whereas modern maize appears to have lost this compensatory mechanism (Gaudin et al., 2011b). However, while teosinte reduces crown root number (CRN) through tillering plasticity under low N stress, modern maize achieves CRN reductions by other means. Other root plasticity strategies likewise differ between teosinte and maize despite a conserved overall response to low-N stress (Gaudin et al., 2011b). Root phenotypic plasticity has important ecological consequences for plant-plant competitive interactions in heterogeneous nutrient environments (Miner et al., 2005); the impact of plasticity on plant-microbe interactions under variable nutrient conditions represents an intriguing future area of study.

Potential tradeoffs between root traits (i.e., acquisition of resources with dissimilar distributions in the soil, possible vulnerability to stresses, costs of investment) (Lynch, 2007) must be considered to determine how the suite of changes observed in modern maize affect performance under different conditions.

## How did maize evolution affect rhizosphere ecology?

Rhizosphere functioning is shaped by the combined influence of host genotype (G), soil environment as affected by agroecosystem management and inherent soil characteristics (E), and their interaction (G × E). Shifts in these determinants during the course of maize evolution resulted in profoundly different selection environments that may have altered ecological functions of the rhizosphere, with consequences for foraging and acquisition of soil resources (Figure 2).

### Impact of directed selection

Maize genotypes differ in recruitment ability, resource provision, and responsiveness to beneficial rhizosphere microorganisms (Kaeppler et al., 2000; Picard et al., 2008; Willmann et al., 2013). This variation in microbe-related traits has a significant heritable component (Peiffer et al., 2013) that could have been acted upon indirectly during selection for aboveground traits of agronomic interest.

Studies comparing teosinte and maize in a single environment suggest a core microbiome has been maintained through domestication, although some rhizobacterial associations may have been lost (Szoboszlay et al., 2015). Strains of diazotrophic *Burkholderia* sp., an abundant genus in the maize rhizosphere, have been isolated from both maize and teosintes grown on an indigenous maize field (Estrada et al., 2002). Similarly, a significant fraction of endophytic bacteria found in teosinte were shown to be conserved in modern maize, and no decrease in endophyte diversity was observed for modern maize when cultivated on indigenous soil (Johnston-Monje and Raizada, 2011; Johnston-Monje et al., 2014). However, rhizosphere bacterial and fungal abundance and activity differed between a teosinte and two modern *Zea mays* varieties, with teosinte having significantly higher bacterial abundance, diversity, and decreased activity of the soil N-cycling enzyme N-acetylglucosaminidase (Szoboszlay et al., 2015).

Domestication also appears to have affected microbiomes of other agronomically important crop species, further demonstrating the potential for human selection to alter host mediation of rhizosphere community structure. The rhizosphere bacterial community of a wild ancestor of beet, *Beta vulgaris* ssp. *maritimis*, has higher diversity, greater resistance to abiotic stress, and a lower proportion of isolates with anti-phytopathogenic activity than that of modern sugar beet (Zachow et al., 2014). The microbiome of domesticated barley (*Hordeum vulgare*) differs from that of its wild ancestors in function as well as diversity, with genes affecting host-microbe interactions showing evidence of positive selection (Bulgarelli et al., 2015). Older and modern cultivars of lettuce (*Lactuca sativa*) have higher rhizobacterial diversity than their wild ancestor *L. serriola*, but diversity indices do not differ significantly between *L. sativa* cultivars (Cardinale et al., 2015).

The impact of modern breeding in a fertile environment on plant-driven rhizosphere determinants has also been observed through “common garden” studies of microbial communities among older and newer maize varieties. Inbred lines from five genetic groups of maize created by human selection were found to support different rhizobacterial communities, especially with regard to the *Burkholderia* genus, but differences were not correlated with genetic distance of the host (Bouffaud et al., 2012). In a subsequent study, however, rhizobacterial community shifts were correlated with phylogenetic distance between maize genotypes (Bouffaud et al., 2014).

The introduction of high-yielding hybrids has had significant effects on the rhizosphere, perhaps because hybrids differ in root traits and exudate production from their inbred parents. Compared to their inbred parents, hybrids generally support more auxin-producing rhizobacteria (Picard and Bosco, 2005); more genetically diverse *Pseudomonas* populations (Picard and Bosco, 2005) and more antibiotic-producing isolates (Picard et al., 2004); stimulate antibiotic production and nitrogen fixation earlier (Picard et al., 2008); and are better at selecting elite rhizobacterial strains (Picard and Bosco, 2006). Heterotic effects have also been studied in AM fungi. A hybrid and one of its parental inbred lines were able to select unique AM fungal communities, whereas the other parental inbred line was not (Picard et al., 2008), suggesting selection of AM fungal strains is controlled by dominant inheritance rather than heterosis. In another study, however, modern hybrids had significantly higher AM colonization than inbreds or landraces (An et al., 2010).

Plants can also affect rhizosphere microbes through changes in provisioning to the rhizosphere (Figure 1). Selecting for high harvest index may have increased aboveground biomass at the expense of exudate quantity and quality, as net rhizodeposited carbon is related to belowground biomass (Amos and Walters, 2006). Altered root traits may likewise have led to changes in the amount, rate, and decomposability of rhizodeposits (i.e., belowground C inputs from root turnover, mucilage, sloughed root debris, exudates), which could affect stimulation of SOM decomposition (i.e., rhizosphere priming) (Kuzyakov, 2002) and subsequent N mineralization (Dijkstra et al., 2009), as well as microbial richness and/or diversity (Bakker et al., 2012). Selective pressure for shifts in microbiome-level metabolism of organic compounds may have been imposed by a decrease in lignin content and lower lignin:N ratio in residues of modern crop varieties as compared to wild ancestors (García-Palacios et al., 2013).

Transgenic approaches to crop improvement have also resulted in substantial changes to rhizosphere inputs (Saxena et al., 1999; Saxena and Stotzky, 2000, 2001); while not strictly the result of breeding, the ubiquity of transgenes in contemporary maize represents a significant alteration of host genotypes that may have had corresponding impacts on the rhizosphere and microbiome (Box 1). Whether changes in resource provision have indeed resulted in altered soil and rhizosphere nutrient cycling patterns remains to be investigated.

In addition to plant-driven effects on the microbiome, changes in plant responsiveness to microbes might have occurred during breeding in high input environments, since plants may derive little benefit from directing C to symbionts in these conditions. If modern maize had an impaired ability to capitalize on beneficial associations for resource acquisition, then growth and yields in low input or biologically-based cropping systems could be compromised. Studies assessing responsiveness to microbial associations in teosinte, early landraces, and modern varieties have attempted to clarify whether domestication and breeding have altered the significance of microbial symbionts in resource acquisition, with conflicting results. No evidence of decreased mycorrhizal responsiveness was found in a comparison of three newer and three older maize cultivars under low-P conditions (Khalil et al., 1994). However, mycorrhizal inoculation caused variable responses in older cultivars, ranging from no effect to 400% higher growth, whereas newer cultivars responded uniformly with higher growth. Similarly, a meta-analysis of 320 crop genotypes found no evidence of decreasing ability to benefit from mycorrhizal fungi over time, with newer cultivars generally less intensively colonized but more responsive (Lehmann et al., 2012). However, mycorrhizal dependence in wheat (*Triticum aestivum*) tends to be higher in landraces than either wild ancestors or modern cultivars (Hetrick et al., 1993) and mycorrhizal responsiveness was lower in modern than older wheat varieties (Zhu et al., 2001). This may indicate that breeding has selected against this trait, perhaps because it was inversely related to phosphorus utilization efficiency (PUtE; Zhu et al., 2001).

### Shifts from natural to increasingly managed soil environment

Soil origin and type appear to be more significant than plant genotype in determining the microbial community structure of the maize rhizosphere (Dalmastri et al., 1999; Gomes et al., 2015) since the rhizosphere microbiome is recruited from bulk soil. However, environmental effects remain poorly understood in comparison to plant-mediated effects, despite the potential for this knowledge to inform agricultural management that creates favorable conditions for biologically-based resource acquisition.

Environmental changes during domestication such as shifting geographic distribution, changes in soil fertility from natural to managed environments, and agricultural cultivation practices influence rhizosphere microbial communities (Pérez Jaramillo et al., 2015). Higher synthetic nutrient inputs and resource homogeneity may have caused the loss of root traits (Milla et al., 2015) and microbial interactions (Wissuwa et al., 2009) that aid in resource acquisition under conditions of lower inorganic nutrient availability. Management practices such as tillage, fertilization, and bare fallows also disrupt the evolutionary stability of mycorrhizal symbioses (Hetrick et al., 1996; Lekberg and Koide, 2005), potentially leading to decreased cooperativity over time (Duhamel and Vandenkoornhuyse, 2013). Quantifying the benefit of microbial associations for maize and teosinte genotypes in wild ecosystems and modern agroecosystems would elucidate whether environmental changes have affected maize-microbe interactions.

### Evolution of genotype × environment interactions

G × E interactions can pose a challenge for breeders and evolutionary studies alike, causing a genotype selected for desirable traits in a favorable trial environment to be poorly suited to variable or suboptimal field conditions (Ceccarelli, 1994). Studying the effect of domestication and breeding on G × E interactions requires the evaluation of multiple genotypes in distinct environments, a study design more frequently employed in nutrient use efficiency studies than microbiome analyses. Mycorrhizal responsiveness showed G × E interactions in four Chinese maize cultivars released between the 1950s and 2008, with the newest cultivar responding positively to colonization regardless of soil P and older cultivars responding neutrally or in a soil-P-dependent manner (Chu et al., 2013). These results suggest that mycorrhizal colonization and responsiveness have co-evolved with other plant improvement traits, although too few genotypes were evaluated to determine whether this is a general trend. Continuing to integrate analyses of microbial responsiveness into resource use efficiency studies will provide useful information on how GxE interactions may affect microbe-mediated resource acquisition pathways.

Phenotypic integration of root and microbial traits may determine the magnitude of G × E-driven evolutionary change in plant-microbe associations (Murren, 2012). Coordination and reciprocal influence between roots and microorganisms are well-characterized, but whether this extends to co-variation over evolutionary time is less clear. Coordinated evolution, i.e., a high level of integration, is predicted to lead to more efficient functioning (Murren, 2012), but may occur to a lesser extent in an environment of artificial selection (Milla et al., 2014). If root and microbe traits are highly integrated, the selective pressures imposed by a heterogeneous, high-nutrient environment may result in evolution toward a rhizosphere where both root and microbe traits are maladapted to low-input systems. In contrast, if root and microbe traits are distinct modules, evolution toward maladaptation to low-nutrient conditions could occur in one module while the other remains unaffected and potentially capable of compensating for lost nutrient acquisition ability.

## What are the implications for agricultural sustainability?

Ever-growing demand for limited natural resources, spurred by population growth and climate change, as well as the high environmental costs associated with conventional agriculture systems requires shifting to management strategies and matched crop genotypes that promote biologically-based resource acquisition over synthetic inputs. Capitalizing on ecological functions naturally present in the rhizosphere, a hot spot of root-microbe interactions, can improve maize productivity in low-input or biologically-based systems and enhance agricultural sustainability in a resource-scarce future.

Recent research has focused on the importance of soil health to agroecosystems (Altieri and Nicholls, 2003), but has failed to account for the central role of the host plant in converting soil health into yield. Rhizosphere microorganisms can reduce the need for external inputs by aiding in the acquisition of scarce resources, but the consequences of microbial community shifts for nutrient cycling and acquisition have been relatively neglected. For instance, understanding whether taxa involved in N cycling and N fixation have been lost or retained and whether any losses have been compensated through functional redundancy would affect not only crop N availability but also rates of N loss from the agroecosystem (Jackson et al., 2012). Similarly, changes in taxa involved in rapid organic matter decomposition or bioavailability of nutrients could affect resource acquisition. Clarifying the genetic basis for loss or gain of microbial traits could pave the way to breeding cultivars that facilitate beneficial microbiomes, thus obviating the need for inoculation to introduce favorable species. Even if changes in microbial community composition are limited, maize genotypes that support high microbial activity and organic matter priming, i.e., where host plant exudates and other rhizodeposits stimulate microbe-mediated nutrient cycling through high exudate quantity and quality, could increase nutrient cycling and utilization of soil resources. Thus, genetic variation in rhizosphere traits must be better characterized and represents a prime target for breeding resource-efficient cultivars.

Modern hybrids show evidence of decreased adaptation to environments of heterogeneous, scarce resource availability, but are better equipped for the acquisition of deep or mobile nutrients (perhaps beneficial under drought conditions) and tolerance of high planting density. Pinpointing the loss of favorable traits on an evolutionary timeline can help identify germplasm for use in creating new, resource-efficient varieties suited to low-input systems. For instance, QTL mapping of teosinte × maize crosses has been used to increase aerenchyma formation (Mano et al., 2007) and similar methods could be used to introgress beneficial allele sources within teosinte and landrace germplasm for increased root hair length or high-affinity ammonium or nitrate transporter expression. Breeding for resource efficiency should be conducted under low-input conditions or where nutrients are supplied from organic sources, so that selection for cultivars able to maintain yields with limited or organic inputs is more efficient (Weber et al., 2012).

Although progress has been made in describing changes in architectural, anatomical, and physiological root traits, as well as microbial community shifts, significant research gaps remain (Box 2). Understanding how human selection has affected root traits and rhizosphere interactions can reintroduce allelic diversity tied to beneficial root traits and microbial associations and inform breeding and management practices that promote biologically-based resource acquisition (Wissuwa et al., 2009).

Box 2 **Knowledge Gaps and Research Needs**

- *Timeline:* How do the roots and rhizosphere microbiomes of early landraces differ from those of teosinte? Root system comparisons of teosinte and modern maize and sequencing studies of older and newer maize cultivars have neglected landraces. Comparisons of root architecture, anatomy, physiology, and ecology along an evolutionary gradient could allow the appearance or loss of beneficial traits and microbial species to be pinpointed.
- *Resources:* How has changed altered plant belowground resource provisioning affected metabolic processes and nutrient cycling in the soil? Metabolomics studies of teosinte, landraces, and modern varieties could provide clues.
- *Responsiveness*: Has maize responsiveness to microbial associations decreased over time? Inoculation studies commonly determine the response of a single maize genotype, but assessing host benefit from microbial associations across an evolutionary gradient could reveal whether responsiveness has declined.
- *Signaling*: How have root exudates and signaling molecules changed? Plant and microbial signaling compounds should be compared across maize and teosinte genotypes; manipulation of phytomicrobiome signaling has been proposed as a strategy to enhance agricultural sustainability (Quiza et al., 2015).
- *Functioning*: What is the functional significance of known changes in community composition for C and N turnover in the rhizosphere? Has plant ability to capitalize on soil health (i.e., sustainable nutrient sources provided by a soil with high microbial activity and diversity) decreased as a result of compositional changes, or has it been maintained by functional redundancy in the microbiome? An extracellular enzyme involved in N cycling differed between maize phylogenetic groups in one study (Szoboszlay et al., 2015), but more detail is needed on nutrient cycling effects and the groups responsible. Sequencing studies can provide only hypotheses based on previous information about the functions performed by a given taxon in isolation, but overlook functional redundancy and interactions. Meta-transcriptomics, meta-proteomics, meta-metabolomics, and novel isotope labeling approaches (Vandenkoornhuyse et al., 2007) could identify key active species and illuminate their functional roles.

## Author contributions

All authors listed have made substantial, direct and intellectual contribution to the work, and approved it for publication.

## Funding

The authors would like to acknowledge University of California and the College of Agricultural and Environmental Sciences for a Graduate Scholars Fellowship to JS and seed research funds from CAES Programmatic Initiative to AG.

### Conflict of interest statement

The authors declare that the research was conducted in the absence of any commercial or financial relationships that could be construed as a potential conflict of interest. The reviewer LC and handling Editor declared their shared affiliation, and the handling Editor states that the process nevertheless met the standards of a fair and objective review.
